# TDCS at home for depressive disorders: an updated systematic review and lessons learned from a prematurely terminated randomized controlled pilot study

**DOI:** 10.1007/s00406-023-01620-y

**Published:** 2023-05-16

**Authors:** Ulrike Kumpf, Ulrich Palm, Julia Eder, Harry Ezim, Matthias Stadler, Gerrit Burkhardt, Esther Dechantsreiter, Frank Padberg

**Affiliations:** 1 Department of Psychiatry and Psychotherapy, Ludwig Maximilian University Munich, Nussbaumstr. 7, 80336 Munich, Germany; 2 Faculty of Psychology and Educational Sciences Ludwig Maximilian University Munich, Munich, Germany; 3 Medicalpark Chiemseeblick, Bernau-Felden, Germany

**Keywords:** Non-invasive brain stimulation, tDCS, Major depressive disorder, Home-based treatment, Home treatment, Remote control

## Abstract

The application of transcranial direct current stimulation (tDCS) at home for the treatment of major depressive disorder (MDD) is the subject of current clinical trials. This is due to its positive safety profile, cost-effectiveness, and potential scalability for a wide outreach in clinical practice. Here, we provide a systematic review of the available studies and also a report on the results of a randomized controlled trial (RCT) on tDCS at home for the treatment of MDD. This trial had to be prematurely terminated due to safety concerns. The *HomeDC* trial is a double-blinded, placebo-controlled, parallel-group study. Patients with MDD (DSM-5) were randomized to active or sham tDCS. Patients conducted tDCS at home for 6 weeks with 5 sessions/week (30 min at 2 mA) anode over F3, cathode over F4. Sham tDCS resembled active tDCS, with ramp-in and ramp-out periods, but without intermittent stimulation. The study was prematurely terminated due to an accumulation of adverse events (AEs, skin lesions), so that only 11 patients were included. Feasibility was good. Safety monitoring was not sufficient enough to detect or prevent AEs within an appropriate timeframe. Regarding antidepressant effects, the reduction in depression scales over time was significant. However, active tDCS was not superior to sham tDCS in this regard. Both the conclusions from this review and the *HomeDC* trial show that there are several critical issues with the use of tDCS at home that need to be addressed. Nevertheless the array of transcranial electric simulation (TES) methods that this mode of application offers, including tDCS, is highly interesting and warrants further investigation in high quality RCTs. Trial registration: www.clinicaltrials.gov. Trial registration number: NCT05172505. Registration date: 12/13/2021, https://clinicaltrials.gov/ct2/show/NCT05172505.

**Graphical abstract:**

*Consider, if feasible to do so, reporting the number of records identified from each database or register searched (rather than the total number across all databases/registers)

**If automation tools were used, indicate how many records were excluded by a human and how many were excluded by automation tools

From: Page MJ, McKenzie JE, Bossuyt PM, Boutron I, Hoffmann TC, Mulrow CD, et al. The PRISMA 2020 statement: an updated guideline for reporting systematic reviews. BMJ 2021;372:n71. https://doi.org/10.1136/bmj.n71. For more information, visit: http://www.prisma-statement.org/

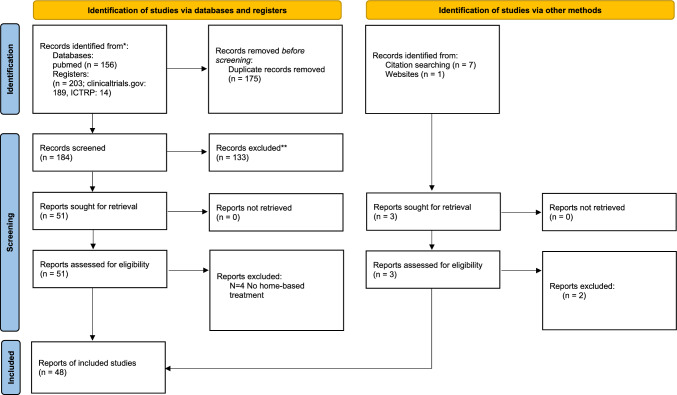

## Introduction

Transcranial direct current stimulation (tDCS) is a technique to non-invasively stimulate the cortex of the human brain and modulate the excitability of cortical neurons by applying a direct current (usually about 1–2 mA). This mechanism of action has been investigated in in-vitro studies, in animal models and in motor cortex studies in humans. Following the leading hypothesis, anodal tDCS may alter resting membrane potentials of cortical neurons by shifting these towards depolarization as well as increasing spontaneous firing rates and cortical excitability, whereas cathodal tDCS exerts opposite effects [1–3]. These activation changes could influence the dysfunctional network activity [4] discussed in the context of psychiatric disorders. In addition, there is evidence for other effects induced by (anodal) tDCS, such as changes in brain-derived neurotrophic factor (BDNF) levels [5], long-term potentiation (LTP) and long-term depression (LTD) via glutamatergic systems [6, 7] and blood–brain barrier permeability [8].

Prefrontal tDCS has been used for many years to treat depressive symptoms. Largely in the context of clinical trials, results are promising, but partly inconsistent [9–12]. Two large RCTs by Brunoni and colleagues [11, 12] demonstrated the antidepressant efficacy of 2 mA prefrontal tDCS for 30 min in comparison with sham tDCS. Meta-analyses and reviews of tDCS show at least moderate effects in the treatment of major depressive disorder (MDD) [13–17]. Overall, non-invasive brain stimulation (NIBS) techniques are promising interventions for the treatment of MDD, particularly for approximately 33% of patients, who are treatment-resistant even after algorithm based pharmacotherapy and psychotherapy [18].

NIBS application is time and personnel consuming, as sessions are daily during the first weeks of acute treatment [15]. The preparation of the sessions themselves take time due to the montage of electrodes and other procedures, and the stimulation itself is 20–30 min per session. For some patients it is not possible to come to the clinic every day. For several years now to address these difficulties, the application of tDCS at home has been proposed and investigated. Especially in times of the COVID-19 pandemic, the home-based treatment approach turned out to be most advantageous, since frequent clinic contact is avoided. In this respect, tDCS may be an ideal intervention, because it involves a small, portable device, is low-cost, and has relatively few side effects [19, 20]. In addition, combination with digital interventions (e.g. gamified training) is possible [21]. So far, tDCS as a home-based treatment has been investigated mainly in the fields of neurology and psychiatry. In fields other than MDD, for example various pain conditions, more than 17 double-blinded randomized controlled trials (RCT) (summary in [22]) with up to 182 patients have been published. The treatment of MDD with tDCS at home has become more intensively investigated in the last few years, however, available studies on this topic consist mainly of case series, monocentric, single-blinded studies or pilot trials. Reasons for this could be the difficulty in requiring patients to take initiative, carrying out and being responsible for a mostly complex treatment on a daily basis. This already highlights one of the first challenges of home-based treatment: ensuring and monitoring treatment adherence. The following systematic review will critically address this and other issues of home-based treatment in a diagnose-specific manner and discuss possible solutions, updating our previous review [23] on the topic of home-based tDCS treatment of MDD. We then present the results of a study that was designed with the critical points highlighted in the systematic review in mind. However, additional problems with this form of application also emerged during trial conduction.

## Review

### Search strategy

The database of the U.S. National Institutes of Health (PubMed/Medline), the WHO International Clinical Trials Platform (ICTRP) and the U.S. National Institutes of Health Clinical Trials Platform (clinicaltrials.gov) were searched without any timeframe (last search on 2022/11/02). The same terms as in the review by Palm and colleagues were used [23] but with the term “depression” or “depressive” added. The terms “tDCS” or “transcranial direct current stimulation” in cross combination with the terms “remote control”, “domiciliary use”, “remotely supervised”, “self-treatment”, “home treatment”, “home”, “self” and “depression” or “depressive” were searched. Furthermore, the terms “do-it-yourself brain stimulation” and “noninvasive brain stimulation remote control”. Database searches (PubMed/Medline) found 156, register searches 203 hits (clinicaltrials.gov: 189, ICTRP: 14). Citation matching brought 7 hits and 1 hit was found via website searching. After manually removing duplicates, 181 hits remained. 133 records were excluded due to topical irrelevance (e.g. no home-based treatment). Some pre-registered studies in the register record had already published results and protocols at time of search. These specific pre-registered studies were excluded to avoid any duplicate hits, as the published results and protocols of these studies had already been included in this review. Pre-registered studies that had been stopped before trial conduction, were also excluded.. 55 abstracts or articles were assessed for eligibility (19 original research papers, 4 published protocols, 6 guideline paper or reviews, 26 trial registrations), 48 remained for analysis.

### Results

The results can be categorized into original research papers, published study protocols, current studies, guideline papers and reviews. With regard to the available original research, mainly case series, pilot studies and open label trials have been published. In some cases, data of newer home-treatment devices of larger cohorts were also read out and analysed naturalistically [24]. Larger RCTs on the topic can be found in the study registers as ongoing or planned studies, and therefore the search results from this area were included to map possible future directions of home-based tDCS in the treatment of depressive symptoms. In addition, the described study designs address current issues such as the combination of home-based tDCS with other (digital or telehealth) methods to augment the tDCS effect. In both ongoing studies/published protocols and original research papers, studies can be divided into those that specifically treat MDD (with varying degrees of treatment resistance, as acute or maintenance therapies) and those that primarily treat other conditions such as chronic pain and rate depressive symptoms as secondary outcomes.

#### Published original research

Of the original research papers identified, 10 papers include results from clinical trials that primarily target depressive symptoms. One real-world study with 452 patients with MDD was excluded from analysis because some patients had tDCS treatment self-administered at home, whereas others underwent tDCS in-clinic settings [24]. A further seven studies show results from home-based tDCS applications for the treatment of other underlying diseases, but with the assessment of depressive symptoms as secondary outcome measures. Table 1 summarizes results of the remaining nine trials primarily targeting depressive symptoms. There are six case series, open label trials or pilot trials and three RCTs with a sham tDCS comparator. The other trials not investigating the antidepressant effects of home-based tDCS as a primary outcome are not listed in Table 1 and include four RCTs and two case series. In addition to the core information for each study, the following parameters listed represent crucial criteria for home-based treatment: (A) control of adherence to the study protocol, (Q) quality of stimulation, including electrode positioning, (S) safety assessment, including reporting of side effects and adverse events, (T) technical monitoring, including storage of stimulation data and (V) regular visits during the study phase to assess clinical changes.

**Table 1:** Summary of characteristics of published original research in the field of home-based tDCS for the treatment of depression

Author, year	Study type, disorder (primary)	Number of participants, age, gender	Conditions Electrode positioning, intensity, duration, number of stimulations	Operator, mode of supervision	Adverse effects, feasibility	Outcome parameters, results	Strengths (S) and weakness (W)	Quality measures during stimulation series A = adherence control Q = quality of stimulation S = safety assessment T = technical monitoring V = regular visits
Alonzo et al. [26]	Open label pilot trial, major depressive episode of a unipolar or bipolar depression, at least 4 weeks	*N* = 34, group 1: *N* = 15, 6 female, 48.64 ± 11.56 years, group 2: N = 20, 7 female, 46.10 ± 13.55 years	Single condition, active: anode F3, cathode F8, 2 mA, 30 min, 20 or 28 sessions in 4 weeks and taper phase of 4 sessions in 4 weeks	Standardized training and checklist in clinic (1 session), Self-administered by patients, remotely observed by research staff via videolink (as needed)	No SAEs Most common side effects: tingling, burning, redness Drop-outs: 2: inability to master set-up, missed sessions. 93% of scheduled sessions completed	Changes from baseline to 1-month follow-up in: MADRS QIDS-SR Q-LES-Q-SF Significant main effect of time in all scales, no effect of session frequency	S: standardized procedures, regular visits and supervision W: no sham condition	A = yes Q = yes S = yes T = yes – electrode contact quality V = yes
Borrione et al. [27]	Case series, open label, combination of home-based tDCS with App based psychological intervention, MDD	N = 5, 4 female, 41.6 ± 9.8 years	Single condition, active + app-based intervention: one-size-fits-all cap, prefrontal, anode left, cathode right, 2 mA, 30 min, 21 sessions in 6 weeks	supervised training, self-administered, remote access to study team in case of any questions or complications	No SAEs All 5 subjects finished the protocol, no drop-outs	Changes from baseline to week 6 in: MADRS, Ham-D-17, BDI-II. 4 treatment responders, 3 remission	S: + app-based intervention. W: no control group, only 5 patients, one-size-fits-all cap	A, Q, S, T in supplementary material, not available. V = yes (baseline, week 2, 3, 4, 6)
Cappon et al. [22]	Open label pilot trial on study companion-administered tDCS at home in older patients with MDD	*N* = 5 (3), two withdrew in the first week, 0 female; 46–72 years	Single condition, active, multichannel tDCS montage with Stimweaver algorithm, F3 (anode), FZ, FC5, and FP1 (cathodes), 1,75 mA max. current, 30 min, 37 sessions in 8 weeks	Study companion-administered after standardized training programme, remote practice sessions, on-demand remote assistance	No SAEs, most frequently reported tingling, itching, sleepiness, scalp redness. 2 drop-outs: medical conditions unrelated to treatment. Of remained patients only one session was missed	Changes from baseline to week 4, 8 and 12 (follow-up) in: MADRS, QIDS-SR16, HDRS, BDI-II. MADRS decreased in all 3 completers (average decrease: 59% baseline to 1-month follow-up), same trend for the other scores	S: new training programme, electrode montage multichannel. W: only 3 patients completed, ratings via phone, no control group	A = yes Q = yes S = yes T = no V = no
Mota et al. [31]	Randomized, double-blind, sham-controlled clinical trial for home-based tDCS in depression in temporal lobe epilepsy	*N* = 26 13 active, 12 female 53.38 ± 14.45 years, 12 sham, 10 female 55.76 ± 7.68 years	Active and sham condition, prefrontal: anode left, cathode right, 2 mA, sham: no current, 20 min, 20 sessions in 4 weeks home-based, then 3 sessions in 3 weeks in-clinic	Self-administered after training by a clinician and with instructional video	7 moderate or severe AEs in active, 3 in sham group: headache, most common side effects were tingling, itching, burning, headache, somnolence, moodswings. Drop-out: 1 (active): pain, burning discomfort. 2 (sham): travel, loss of interest	Changes from baseline to week 2,4, 8 in the BDI-II (primary Outcome), QOLIE-31 and HAM-A score (secondary Outcome), improvement over time but no group difference	S: active and sham, monitoring of technical parameters. W: different groups of medications	A = yes Q = yes S = yes T = yes V = yes
Sobral et al. [28]	Case series, open label, combination of home-based tDCS with App based psychological intervention, MDD and/or comorbidities	N = 7, 4 female, 26–51 years old;	Single condition, active + app-based intervention: one-size-fits-all cap, prefrontal, anode left, cathode right, 2 mA, 30 min, 18 or 21 sessions in 6 weeks	Supervised training, self-administered by patients, clinical progress monitoring in person and remotely using Zoom	No SAEs, most common AEs: scalp irritation, tingling, itching, and burning sensation. Adherence and acceptability overall high (ACCEPT-tDCS scores), 2 patients missed > 50% tDCS sessions (personal challenges), 1 drop-out (compliance)	Changes from baseline to week 6 in: MADRS-Self rating and BDI-II, STAI-Trait clinical improvement in MADRS-S in 5 patients and in STAI-Y2 5 patients	S: combination with app-based intervention W: 2 patients initiated CBT at same time as study, no control group	A = yes Q = no S = yes T = no V = yes
Woodham et al. [30]	Open label, single-arm study of home-based tDCS in MDD	*N* = 26, 19 female, 40.9 ± 14.2 years	Single condition, active tDCS, F3 anode, F4 cathode, 2 mA, 30 min, 21 sessions in 6 weeks	Self-administered, research team member was present in person or by real-time video call at each session	No SAEs, most common side effects were skin redness, tingling, itching, mild burning sensation, headache. Acceptability rated high, drop-outs: 3 (broken device, physical health, personal reasons), 92,8% completed 6 weeks of treatment	Changes from baseline to week 6 and after 3 and 6 months follow-up in: HAMD. Response in 22 (20) participants, clinical remission in 21 (18) at week 6 (after 6 months)	S: 26 patients included, long follow-up phase, Acceptability questionnaire. W: no control group	A = yes Q = no S = yes T = no V = yes
Le et al. [29]	Case series, retrospectively examined clinical data, MDD	*N* = 16, no further information, only abstract available	Single condition, active tDCS, different protocols, up to 2 years of treatment, at least 6 weeks	Self-administered at home	No SAEs, 2 patients withdrawn because of blurred vision or exacerbation of tinnitus	5 patients responded to acute tDCS within 6 weeks, 9 patients who received tDCS for more than 12 weeks maintained improvements over several months	S: long observation period W: no control group, active group very inhomogenious	No further information
Oh et al. [32]	Randomized, single-blind, sham-controlled clinical trial, parallel group, MDD, home-administered tDCS + Escitalopram 5–20 mg/d	*N* = 58, 29 (20) active, 29 (25) sham, 9 drop-outs in active, 4 in sham group, Active: 10 female, 29.7 ± 11.6 years, sham: 10 female 28.5 ± 11.0 years	Active and sham condition, anode F3, cathode F4, active: 2 mA, sham: no current, 30 min, 30 sessions in 6 weeks	Self-administered at home, instruction once by a research nurse, online manuals	2 skin burns (forehead) Drop-outs: 13 (9 active: 2 burns, 7 poor treatment compliance; 4 sham: 4 poor treatment compliance)	Changes from baseline to week 6 in: MADRS, HAM-D, BDI. Improvement in all scales over time, BDI score decrease was significantly different between the active and sham tDCS groups	S: active and sham condition, regular visits. W: single-blinded design	A = yes Q = no S = yes T = yes (current density) V = yes
Lee et al. [33]	Randomized, double-blind, sham-controlled clinical trial for home-based tDCS in bipolar depression	*N* = 64, 47 female, 33.4 ± 12.6 years	Active and sham condition, anode F3, cathode F4, active: 2 mA, sham: no current, 30 min, up to 42 sessions in 6 weeks	Instructions, self-administered at home, video training, on-demand video or voice calls	No SAE, most common side effects were headache, skin redness, tingling. Drop-outs: 26 (13 active: 8 poor compliance < 60% scheduled sessions, 3 withdrawals, 2 of them due to AE; 10 sham: 7 compliance < 60% of scheduled sessions, 1 AE, 1 patients request, 1 non-compliance)	Changes from baseline to week 2,4, 6 in the HDRS-score between groups over time time-group interaction for the HDRS-17 were not statistically significant	S: active and sham condition, regular visits. W: no follow-up	A = yes Q = no S = yes, only every 2 weeks T = no V = yes

*MADRS* Montgomery Asberg Depression Rating Scale, *QIDS-SR* Quick Inventory of Depressive Symptomatology self-report, *QLESQ-SF* Quality of Life Enjoyment and Satisfaction Questionnaire–Short Form

To our knowledge, the first research report on home-based tDCS for the treatment of depressive symptoms was a case report published in 2018 (not listed in Table 1; [25]). In 2019, Alonzo and colleagues published the first open label pilot trial specifically investigating the use of home-based tDCS in a depressed sample [26]. Two recent case series of interventions involving tDCS combined with app-based behavioural therapy reported antidepressant effects, feasibility and safety.[27, 28]. Some problems of adherence, with only one patient completing the course per protocol, were also reported by Sobral and colleagues. For a group of elderly patients with MDD, Cappon and colleagues established a study companion, who was trained in the patient’s place and carried out the treatment for them at home. In this study, problems with adherence were also reported (i.e. only 3 out of 5 patients continued treatment beyond the first week) [22]. Another case series in treatment-resistant MDD [29] and an open label single-arm study [30] confirmed the feasibility, safety, and antidepressant efficacy of home-based tDCS in 16 and 26 patients, respectively.

Further three RCTs were reported [31–33], the only three placebo-controlled studies published in this field to date, one treating depression in bipolar disorder [33], and another depression in temporal lobe epilepsy [31]. A significant improvement in depressive symptoms over time, but without a difference between groups, was reported for two double-blind RCTs with 64 and 26 patients, respectively [31, 33]. Concomitant pharmacotherapy was miscellaneous including mood stabilizers, antiepileptic drugs, and other classes of medications and therefore not necessarily comparable to antidepressant medication standards.

Oh and colleagues showed a significant improvement of depressive symptoms (according to self-rating, but not observer rating) in a single blinded, sham-controlled RCT, however 13 out of 58 patients did not complete the study [32].

In addition, we found eight studies that evaluated depressive symptoms not as a primary outcome but as secondaries, as these studies focussed on other target symptoms and conditions. Two cases of patients with amyotrophic lateral sclerosis (ALS) showed no improvement of depressive symptoms [34]. Another study investigated the effects of home-based tDCS in 20 [35] and 48 [36] fibromyalgia patients on symptoms of pain and pain-related disability. For active tDCS, a significant improvement of these symptoms and concomitant depressive symptoms was observed. Another open label trial failed to demonstrate any antidepressant effect over time after 10 self-administered tDCS sessions in older patients with knee osteoarthritis, but both clinical pain severity and sleep disturbances significantly improved [37]. Another RCT investigated the effects of tDCS in patients with chronic pain who had previously responded to rTMS. No significant improvement of depressive symptoms was observed, but a change pain levels by 15% [38]. Another open label pilot trial used prolonged exposure of self-administered tDCS over the motor cortex in 21 veterans with posttraumatic stress disease (PTSD) to treat chronic pain and symptoms of PTSD. This study found a significant improvement for both and a trend towards improvement of depressive symptoms, but no change in pain intensity [39]. One RCT investigated the efficacy of prefrontal, self-administered tDCS on migraine symptoms. No significant difference was shown between the sham and active groups for the number of migraine days (primary outcome) or for the co-assessed depressive symptomatology [40].

#### Current clinical trials and published study protocols

A total of four very innovative protocols on home-based tDCS for the treatment of depression have been published. One of them [41] has already reported results and is therefore discussed above [38]. Another protocol describes a parallel-group RCT (double-active vs double-placebo), combining home-based tDCS and cognitive control training in the treatment of 114 MDD patients. In addition to investigating the synergy of tDCS and mini games for cognitive control training, an interesting aspect of this study is the implementation of an electrode-positioning algorithm that allows the correct positioning of the electrode cap even during self-application at home [21]. Another protocol describes a large RCT with 210 patients and three arms (70 each), where home-based tDCS is investigated in conjunction with internet-based behavioural therapy (iBT) [42]. The third protocol describes a RCT focussing on the long-term use (i.e. over 6 months) of home-based prefrontal tDCS in 100 patients with Alzheimer’s disease. This parallel-group design is split into active tDCS and sham tDCS with 50 patients planned per group. The primary outcome is cognition, depressive symptoms secondary [43].

While searching clinicaltrials.gov and ICTRP, a large number of abstracts for current trials were found on the topic. For some studies only sparse information is available. Abstracts can be accessed via the webpage https://clinicaltrials.gov/ct2/search and are not mentioned in the reference list. A total of 26 abstracts were found and screened. These are large, partly multicenter RCTs investigating the effect of tDCS in neurological diseases (Huntington`s disease, multiple sclerosis, migraine) or chronic pain, often combined applications with exercise, where depressive symptoms are described at most as a secondary outcome. Another study plans to investigate home-based tDCS against apathy in Alzheimer`s disease, apathy being a new target symptom. There are also some psychiatric studies, where depressive symptoms are not primary but secondary outcomes; i.e. a study on home-based tDCS for treating suicidal ideation, another on craving in cocaine addiction, one study on behavioural symptoms in Alzheimer`s disease, one study in elderly depressed patients, and a RCT on home-based tDCS for treating self-harming behaviour. Other planned or ongoing studies investigate treatment of peri-partum depression, e.g. an open study combining home-based tDCS with an e-health application for postpartum depression. In addition, a small sham-controlled study on home-based tDCS for peri-partum depression in pregnancy is planned. Overall, the home-based and safe application of tDCS would be of decisive advantage for treating per-partum mental health conditions. Another trial on home-based tDCS for enhancing cognition in mildly cognitive impaired patients with late life depression has been planned as an open label trial. Three RCTs investigate the application of home-based tDCS for the treatment of unipolar depression (including the *HomeDC* trial), another RCT spotlights treatment-resistant depression.

Four further studies do not include a control group, most of them testing new devices for home-based tDCS. One study will investigate home-based tDCS in bipolar depression. Of particular interest are two abstracts about ongoing studies focussing on home-based tDCS as maintenance therapy, one after rTMS (responders only) and the other after successful ECT in conjunction with computerized cognitive behavioural therapy. The rTMS trial is placebo-controlled and the ECT trial is an open label study. Both studies address the important research question of whether home-based tDCS for depression is effective as a maintenance therapy after neuromodulatory interventions.

#### Guideline papers and reviews

Six reviews and guideline papers on the topic of home-based tDCS were found; specifically on the topic of home-based tDCS for the treatment of depression. No summary publication is yet available. The guideline papers focus on the following points, which are considered important to varying degrees depending on the paper for the home-based use of tDCS [44–49]:

- criteria to evaluate if patients are suitable to perform tDCS remotely
- Electrode positioning (should be easy and replicable but with respect to individual anatomy)
- Monitoring of adherence
- Training and supervision of patients, training material, supervision on-demand or always. Troubleshooting if problems occur during stimulation at home.
- Monitoring of stimulation quality (technical parameters, dose control)
- Safety monitoring, guidelines for discontinuation of a session, monitoring for treatment-emergent adverse effects
- Blinding in clinical trials

The issue of electrode positioning is discussed in detail in a review by Borrione and colleagues [48] on the subject of the development of a home-based tDCS device. On the one hand, e-field modelling shows that a shift of the electrodes by 10% of the distance between nasion and inion already leads to relevant changes in current density and e-fields. This would be quite likely in the case of a one-size fits all solution due to different head sizes and anatomies. On the other hand, the intensity of the e-field determines the dose that arrives at the dorsolateral prefrontal cortex and there is still no clear statement about the best dose for different treatments, for example of depression. Meaning this aspect could become invalid through different treatment protocols. Specifically to the treatment of depression with home-based tDCS, it is stated [45] that suicidality plays a major role and must be monitored intensively. This is also due to the danger of an increase in motivation in the early treatment phase, which can increase the risk of suicide. As a result, technical control of the device is also important, so that it cannot be used for deliberate self-harm. It should also be closely monitored whether the patient is able to adequately perform the tDCS treatments. In case of an additional deterioration, a contingency plan should be developed. In patients with depression, the suitability for home-based tDCS should be thoroughly checked. They may be quickly overburdened by their depressive symptoms, possibly due to concentration problems, or have too little energy to carry out the treatment themselves. Concomitant medications should be clearly monitored and benzodiazepines/anticonvulsants should be avoided in trials due to their clearly expected impact.

## Summary

In summary, the review presents the current state of research on home-based tDCS for depression. The published original papers show a trend towards good antidepressant efficacy. Although according to most of the few sham-controlled studies, there is no significant difference between placebo and active stimulation. Furthermore the protocols vary strongly with different dosages and lengths of treatment, ranging from a few sessions to years, and are therefore poorly comparable. Our handful of RCTs mostly stayed true to the same regimen: a 6-week treatment, about 30 stimulations, and an optional maintenance phase. This is because similar protocols yielded the best effects during in-clinic tDCS trials on MDD. However, adequate dosage is not only dependent on duration, frequency and total number of stimulations, but also on exact electrode positioning [48]. E-Field studies have shown that this is inconsistent across studies due to use of different devices. We are seeing more innovative approaches appearing in published study protocols and current trial abstracts. This includes combining tDCS with psychological interventions or the use of home-based tDCS as a maintenance therapy after ECT or rTMS. TDCS is also being extended to special cohorts such as pregnant patients. Overall, however, there is a lack of placebo-controlled RCTs to properly assess the efficacy of home-based tDCS in MDD. Larger cohorts are needed to better demonstrate trends in efficacy and significance.

The study presented below provides a contribution to the investigation of the feasibility, safety, and effectiveness of home-based tDCS for depression. It critically highlights relevant issues in home use, while opening up new possibilities in monitoring and electrode positioning.

## Methods

### Trial design and study objectives

The *HomeDC* trial is a double-blind, placebo-controlled, parallel-group study with 16 patients per group (trial registration number: NCT05172505, clinicaltrials.gov). The study has been approved by the local ethics committee of the Ludwig-Maximilians-University Munich (project-number 21–0731) and the trial was strictly conducted in accordance with the Declaration of Helsinki. Written informed consent was obtained from all patients prior to participation. Sample size and power calculation was based on the effects in the unimputated ITT sample of the SELECT trial [11]. The study was conducted at the Department of Psychiatry and Psychotherapy of the LMU Munich. Patients with a primary MDD diagnosis were randomized to either active or sham stimulation groups. The patients then self-administered a maximum of 30 prefrontal tDCS sessions (active or sham) over the course of 6 weeks. The stimulations were either a monotherapy or an adjunctive treatment to stable antidepressant medication. Furthermore the sessions were only permitted on work days. Depressive symptoms were rated by an independent rater and via self-rating scales at baseline and after 1, 2, 4, 6 and 10 weeks. As only 2 patients completed the initially planned 14-week follow-up visit, the results will focus on the other visits.

Feasibility was the primary outcome, and it was evaluated according to the number of completed stimulation sessions and drop-out rates. Safety (secondary outcome) was evaluated based on the number of (serious) adverse effects. Antidepressive effect was measured using the change in the Montgomery and Asberg Depression Rating Scale* (*MADRS), the Beck Depression Inventory (BDI), the General Assessment of Functioning (GAF) and the Clinical Global Impression-Improvement/-Severity (CGI-I/-S) scores over time; as well as between the active tDCS and sham tDCS groups. Stimulation quality and adherence was evaluated based on stored and transmitted technical stimulation data.

### Inclusion and exclusion criteria

Men and women between the ages of 18 and 70 with a primary diagnosis of unipolar major depressive episode according to DSM-5 criteria with a total score of ≥ 13 in the Hamilton Depression Rating Scale (HDRS-17 (Hamilton, 1960) at the screening visit were recruited at the Department of Psychiatry and Psychotherapy of the LMU Munich (episode single or recurrent, duration of current episode at least 4 weeks but no longer than 5 years). Patients either had no medication or were on stable medication for at least 2 weeks (3 months for Lithium) prior to inclusion (Benzodiazepines and Zopiclone only as rescue medication limited to 7.5 mg and 2 mg Lorazepam dose equivalent respective). Patients with any relevant psychiatric axis-I- and/or axis-II-disorders as a primary diagnosis other than MDD or any relevant neurological disorders (including history of seizures) were not included. Patients were naïve to tDCS with the exception of single tDCS sessions during experimental studies. Concomitant psychotherapy was permitted. Type, modality (e.g. group vs. individual therapy), duration and frequency of therapy during study participation was documented. Exclusion criteria included acute risk for suicide, ECT in the current episode, intracranial implants and known or suspected pregnancy (according to pregnancy test at baseline visit).

### tDCS procedure and blinding

Patients self-administered a maximum of 30 daily (work days) prefrontal tDCS sessions over 6 weeks. Electrode montage was bifrontal with the anode over F3 and the cathode over F4 (international 10–20 EEG system). Stimulation was 2 mA in the active condition, with a duration of 30 min each, plus ramp-in (15 s.) and ramp-out (30 s.) phases at the beginning and end of treatment. In the sham condition the same ramp-in and ramp-out parameters were used but without intermittent stimulation.

We used CE-certified neuroConn DC-stimulators, which allowed for measurement, recording and transfer of technical stimulation data (impedance, voltage and current) every second of stimulation. Impedance was tested using a test current prior to each stimulation. An automatic mechanism either stopped the stimulation at impedances above 55 kOhm or did not allow the stimulation to start (analogous to the DepressionDC trial [50]). An automatic lock prevented any repeat stimulation for the first 16 h after the last stimulation. For correct electrode positioning a CE-certified cap (neuroConn) with the electrodes already implanted was used. The appropriate size, out of five different sizes, was chosen not only according to the head circumference, but also in relation to the distance between the vertex and external eye angle allowing a more precise electrode positioning at the F3 and F4 points. Saline was used as contact medium (20 ml per sponge). Blinding and randomization procedures were performed analogously to the *DepressionDC* trial [50] ensuring a blinding of investigator, rater and patient.

### Safety monitoring

Patients completed the Comfort Rating Questionnaire (CRQ), a self-report questionnaire on side effects of brain stimulation [51], after each session (paper–pencil) and were instructed to call the study team if an unusual event, pain or other problem occurs. During the biweekly ratings in the clinic, patients were also specifically asked if any AE occurred.

### Statistical analysis

The data were evaluated using the software “IBM SPSS Statistics 29”. To analyse group effects (active vs sham, differences in total scores of MADRS, BDI, CGI and GAF), time effects (baseline, week 6 [V4, i.e. post stimulation] and week 10 [V5, i.e. follow-up]) and their interaction, we used a 2 × 3 mixed analysis of variance (ANOVA) with group as a between factor and time as a within factor.

## Results

### Participants

The study originally planned to include 32 patients (16 per group). Due to early termination of the study because of an accumulation of skin burns, only 11 patients (5 active) were recruited. Characteristics of the study population are listed in Table 2.

**Table 2:** Mean baseline demographic and clinical characteristics of MDD participants, Mean values are presented with standard deviation in parenthesis

	Active	Sham	All (active = 45%)
Total number (female)	5(1)	6(3)	11(7)
Age (at baseline)	35.80(10.83)	38.67(13.62)	37.36(12.51)
Years of education	14.40(2.87)	15.83(5.49)	15.18(4.55)
Duration of illness (years)	5.14(3.28)	6.28(2.79)	5.76(3.08)
Duration of current episode (weeks) (range)	75.80(61.51) (12–171)	179.83(83.22) (119–271)	132.55(90.44) (12–271)
Previous number of episodes	3.50(3.35)	1.00(1.22)	2.25(2.82)
Number of tDCS sessions	24.20(5.64)	26.50(5.53)	24.45(5.69)
Baseline HDRS	19.20(2.48)	21.50(3.59)	20.45(3.34)
Baseline MADRS	22.00(4.15)	24.33(5.59)	23.27(5.129
Number of adequate trials in current episode	3.80(1.33)	3.33(4.38)	3.55(3.37)
Current psychotherapy (yes in %)	80%	66.67%	73%

### Feasibility

The feasibility of home-based tDCS was descriptively evaluated. Due to a mistake with the codes by the investigator, only 15 stimulations could be performed in patient HDC09. As this was an error by the study team, the patient is excluded from the analyses. Despite this singular case of code confusion, the procedure of codes being entered for either active or sham conditions, adopted from the DepressionDC study, proved to be feasible.

The remaining 10 patients (5 active) performed an average of 26.5 stimulations during the 6 weeks, which corresponds to the average number of 26 stimulations targeted in the protocol. The number of stimulations did not differ significantly between sham (mean 28.8) and active (mean 24.2) patients, leading to overall feasibility being considered good.

### Safety

The study was stopped due to five AEs in four patients (one patient had two AEs). All five AEs were skin lesions and occurred in patients in the active group, which means that four of the five patients in the active group had a skin lesion. Based on the impedance measurements, no clear user misapplication could attributed to the cause of the skin lesions.

The safety of the study can thus be described as inadequate. There were 280 regular stimulations performed until discontinuation. Five of which resulted in an AE (corresponding to 1.8% of all stimulations). Safety monitoring (as described above) was found to be inadequate in this case. There were no consistent indications in the CRQs beforehand, such as an increase in pain. Only one of the patients (HDC04) reported increased burning (5/10) and increased pain (4/10) in the CRQ. However values of 4 and 5, in “burning” and “pain” respectively, were already reported by the patient in some of the CRQs of previous stimulations that had not led to an AE. Apart from one patient, who did not notice anything at all, the other patients retrospectively reported a brief and intense burning sensation during the corresponding stimulation, which, however, disappeared so quickly that the stimulation was not interrupted. One patient described a light flash during stimulation, another patient retrospectively described an increase in the sensitivity of the skin. This increase in skin sensitivity for about five days before the lesion occurred was explicitly not described as painful and burning. Furthermore, two of the four patients failed to contact the study team by themselves after the AE. Their lesions were instead noticed during the mandatory inspection routine of the regular study visits. One patient even went on to put a small patch over the wound and continued stimulation for two more sessions before the next study visit. All despite each patient being individually instructed and periodically reminded to call the study team if any unusual event, pain or problem were to occur. The lesions all healed completely.

### Antidepressant effects

To measure antidepressant effects, the MADRS was used as an investigator-based rating scale, and the BDI as a self-rating scale. In addition, the CGI and the GAF were assessed. Comparisons were made between groups (active vs. sham) and between assessment time points using a 2 × 3 mixed ANOVA with group as a between factor and time as a within factor.

Table 3 descriptively summarizes the antidepressant effects (time effects) for both groups. As can be seen, both groups improved on most variables with a slight advantage for the active group from baseline to post-treatment; an advantage no longer there at the follow-up assessment.

**Table 3:** Mean values in clinical scales over time for active and sham group

Dependent variable	Baseline		Post-treatment		Follow-up	
	Active	Sham	Active	Sham	Active	Sham
MADRS	22.0	24.8	13.0	19.6	17.3	17.3
BDI	26.6	29.4	20.0	21.2	19.8	21.0
CGI	4.0	4.8	3.4	3.2	4.0	3.8
GAF	53.0	48.6	63.0	59.0	60.0	58.8

**Fig. 1:**
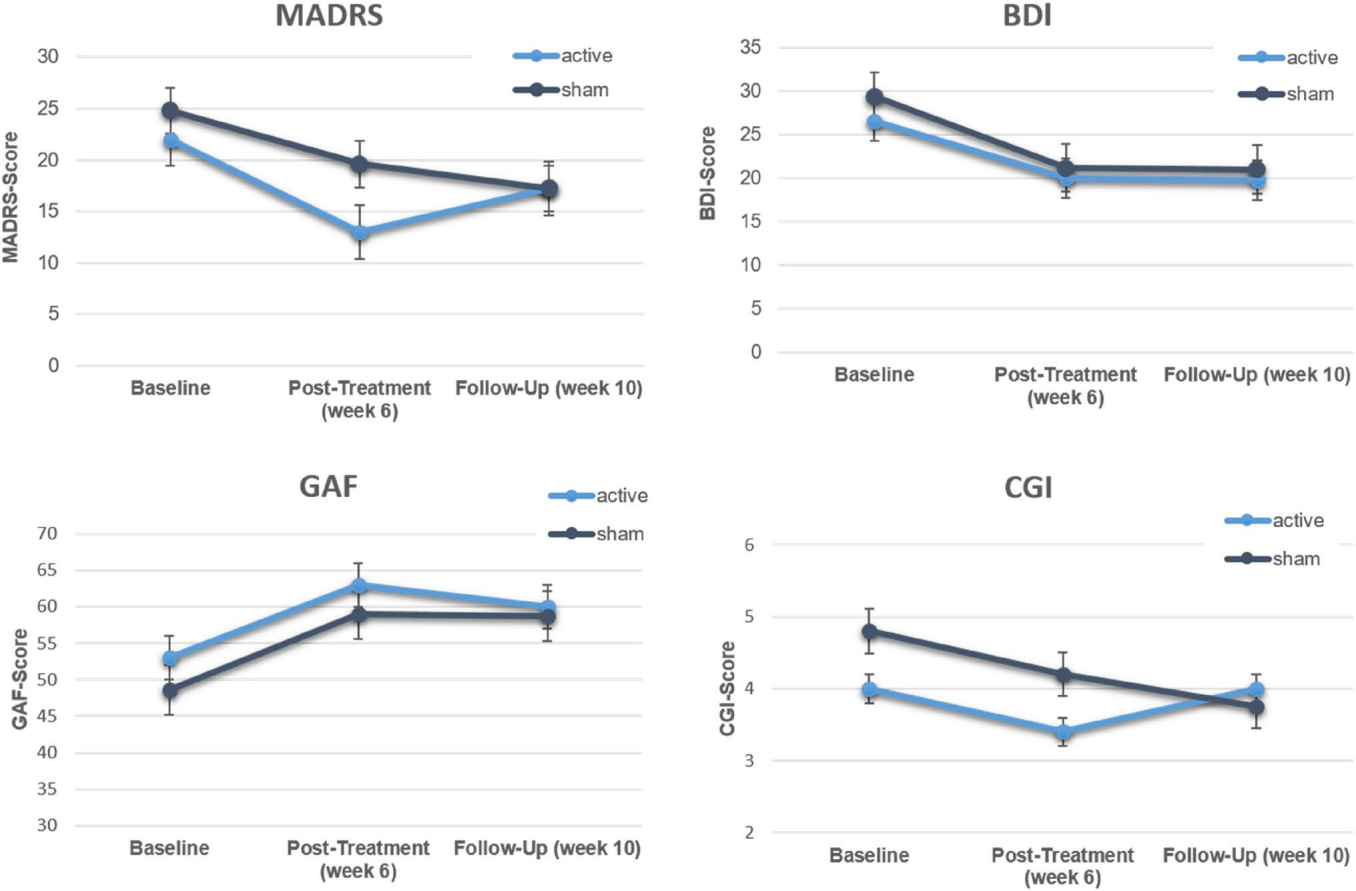
MADRS-, BDI-, CGI- and GAF-Scores at baseline, week 6 (post-treatment V4) and week 10 (follow-up V5). *Consider, if feasible to do so, reporting the number of records identified from each database or register searched (rather than the total number across all databases/registers). **If automation tools were used, indicate how many records were excluded by a human and how many were excluded by automation tools. From: Page MJ, McKenzie JE, Bossuyt PM, Boutron I, Hoffmann TC, Mulrow CD, et al. The PRISMA 2020 statement: an updated guideline for reporting systematic reviews. BMJ 2021;372:n71. https://doi.org/10.1136/bmj.n71. For more information, visit: http://www.prisma-statement.org/

The inferential statistics further corroborate this picture. There was a significant time effect for MADRS (*F*_2;12_ = 12.56; *p* = 0.001; *η*^2^ = 0.19), but neither a significant group nor an interaction effect. Similarly there was a significant time effect for the BDI (*F*_2;12_ = 8.24; *p* = 0.006; *η*^2^ = 0.13), but neither a significant group nor a interaction effect. The same pattern of results was observed for the GAF with a significant time effect (*F*_2;12_ = 5.77; *p* = 0.018; *η*^2^ = 0.22), but neither a significant group nor an interaction effect. There were no significant effects for the CGI. Figure 1 illustrates the four analyses visually.

## Discussion

Based on the results of the systematic review, the *HomeDC* trial, a pilot RCT, was set-up with the plan of a multicenter RCT in mind. The trial was to take the groundwork laid out by previous findings into consideration. This groundwork included a new way of monitoring technical parameters, adequate blinding, as well as adherence and safety monitoring/maintaining mechanisms. Additionally, a new method of electrode positioning was to combine the highest possible accuracy with ease of use. However despite these considerations, the implementation of the *HomeDC* trial showed difficulties, especially with regard to safety, leading to its premature termination.

The results of the *HomeDC* trial help us draw the following conclusions in regard to safety in a home-based tDCS treatment setting:

- Monitoring technical parameters (i.e. impedance) is not enough to prevent AEs like skin lesions (1)
- Patients must be instructed in a clearer more standardized manner (2)
- Patients must be more closely monitored (3)
- The equipment (caps) used may have contributed to the skin lesions (4)

(1): It has become apparent that monitoring technical parameters alone, for example impedance is not sufficient enough to prevent AEs such as skin lesions. This is especially the case if the technical parameters are not monitored online with real-time data transfer. During the *HomeDC* trial, data on the quality of individual stimulations was analysed by an external investigator, usually one to several days after the stimulation occurred. A lack of immediate feedback thusly made a prompt reaction to occurring AEs impossible. Although subsequent monitoring can identify technical defects and user errors (pulling on the cables during treatment or too little sodium chloride solution) as likely or unlikely causes for AEs after the fact; simultaneous online monitoring would prove useful in preventing AEs by detecting real-time increases in impedance and voltage, sending an automatic safety message to the study team in case of any change.

(2) and (3): The patients’ behaviour also shows us that instruction must follow in a clearer and more standardized manner. Closer monitoring is advisable, for example in the form of video calls. Furthermore, expecting patients to reach out if they have any problems is not reliable, as they often failed to do so despite clear communication of the research team’s active availability. Possible solutions involve patients filling out a safety questionnaire before and after stimulation, which would explicitly examine the condition of the skin; not only felt sensations (CRQ). The feedback results would then be directly transmitted to the research team. Careful and active monitoring seem to play a role in the treatment of depressed patients in particular, as they may not be able to proactively report any abnormalities or problems themselves due to their depressive symptoms.

(4) The equipment used, namely the caps, may have contributed to the occurrence of skin lesions. Cases of skin lesions after tDCS are quite rare [49], but there are still singular reports. Suspected causes in the reported cases were [51–53] the use of tap water possibly causing higher impedances, [51, 54, 55] the use of electrode gel or electrode cream with uneven distribution causing increased impedance, [56] certain skin problems pre-stimulation, [57] and insufficiently moistened electrodes. Of the possible causes reported, only insufficient moistening of the electrodes could be considered as a cause for the skin lesions in the *HomeDC* study. Consequently, insufficient moistening would result in increased impedance leading to the skin lesions. However, first analyses show that in the *HomeDC* study no impedance breakouts were found in the corresponding cases. In this respect, further analyses are still required. The experiences gained lead to the recommendation, that no newly developed equipment, including CE-certified equipment, should be used for the first time in a home-treatment environment. Rather it should first be tested for its long-term clinical use, establishing close monitoring of controls with any irregularities being reported directly and at a low threshold to the on-site treatment team. Inspection of the skin would be carried out before and after stimulation by the operator. After the first case of a skin lesion during the trial, steps towards prevention in all other participating patients were taken, in the form of re-training sessions with one additional on-site supervised stimulation. In the re-training session, safety aspects, necessity of skin inspection and the importance of correct montage were explicitly explained again. The patients were also reminded that they were allowed to interrupt a stimulation. The findings above may explain why efforts made after the first case to find any “mistakes” and stop any further skin lesions from occurring were unfruitful. Results of secondary outcome parameters of the *HomeDC* trial show that home-based tDCS induces antidepressant effects, however, no differences were found between active and sham tDCS. Thus, the results are in line with previous studies, even though only one placebo-controlled study applied home-based tDCS specifically to MDD patients [32]. In this trial, the self-report questionnaire BDI showed a significant superiority of the active group over the sham group. All other scales failed to demonstrate any group differences. However, the very small sample size majorly limits any further interpretation. The premature termination of the *HomeDC* trial, due to the presence of multiple skin lesions resulted in the inclusion of a cohort (*N* = 11) far below the power analysis target (*N* = 32). Individual trends, such as the decrease in MADRS at the post-treatment time point, were more marked in the active group than in the sham group but did not indicate a significant difference between both groups. Nonetheless the identified group difference had a high effect size (Cohen’s *d* =  − 0.99) and may have become significant had a higher power analysis target been achieved. Further RCTs with larger patient cohorts are needed.

Overall, the problems reported in the *HomeDC* study reflect current problems in the field. These are also considered and reported in the above review. In home-based tDCS treatment and related studies, special attention must be paid to the following issues:

- Training and supervising patients
- Anatomically individualized electrode positioning
- Blinding
- Troubleshooting difficulties during at home stimulation
- Monitoring stimulation quality, safety, and AE development
- Monitoring adherence (especially considering the nature of symptoms in MDD patients)

The *HomeDC* trial attempts this, but fails in safety monitoring. Conceivably giving future trials an indication of how to better implement safety concepts. On the topic of safety monitoring, systems should be established with real-time monitoring combined with real-time feedback online. A balance must be found between personnel costs and a good safety concept; possibly achievable with machine-based, app-based processes and automatic, real-time feedback.

The review highlighted that the field of home-based tDCS remains open. These four areas in particular:

1. TDCS home treatment in special patient groups, who have more difficulty coming to the clinic than the normal collective. Such as pregnant people or postpartum individuals. This review revealed projects both ongoing and in development.
2. Long-term home treatment as a maintenance therapy, possibly after ECT, TMS, or successful psychotherapy. Here it will be a question of frequency, indication, and discontinuation. More clarification on tDCS effectiveness in this capacity is also needed, although individual case reports are positive [58]. The review section indicates mentionable studies being set-up in this field as well.
3. Furthermore protocols that are more difficult to implement in the clinic due to a higher time requirement could be tested and established in a home-based treatment setting, potentially resulting in increased side effects. An example could be stimulation sessions several times a day, analogous to rTMS and the Stanford Accelerated TMS protocol (SAINT).
4. The combination of tDCS with other interventions could also be tested in the home-based setting. This is expected to have a synergistic effect, feasibly improving the rather small effects of tDCS compared to placebo [21]. A RCT examining the combined effects of tDCS and behavioural therapy in a clinical group setting had negative results, however this study lacked a double-placebo condition [59]. In the future, it would be important to know which interventions are effective and complementary to tDCS determining the optimal time to achieve the greatest effects possible: In intervals, before, during, or after tDCS.

Further future perspectives include other stimulation forms like transcranial alternating current stimulation (tACS) in home-based settings and a combination with neuroimaging procedures after multiple sessions (i.e. after long-term tDCS treatment).

## Conclusion

The systematic review on the topic of tDCS at home for depressive disorders provides an overview of the current data available. It revealed that there are only three RCTs [31–33] on the topic, each with rather small samples, while the majority of publications on the topic are not placebo-controlled. Amongst the three RCTs, except for one study in terms of the BDI [32], the active group was not superior over the sham group regarding antidepressant efficacy. However, the generally small number of RCTs and small sample size should leave definitive conclusions open. Furthermore, the review highlights the problems and challenges of home-based tDCS application in depressive disorders, also echoed in the reported study. In this respect, the conclusion that home-based tDCS is safe for use in depressive disorders, cannot be adopted for the current study. We hope that the lessons we have learned from the HomeDC trial may help as caveats in future studies to improve safety and feasibility of tDCS application at home. Our results demonstrate the need for establishing sound safety concepts for further studies with home-based tDCS and other tES methods, as well as to strive for the recruitment of larger samples.

## Data Availability

The data that support the findings of this trial are not openly available due to reasons of sensitivity and are available from the corresponding author upon reasonable request. Data are located in controlled access data storage at University Hospital Munich, department of psychiatry and psychotherapy.
